# β-blockers and statins: exploring the potential off-label applications in breast, colorectal, prostate, and lung cancers

**DOI:** 10.3389/fphar.2024.1423502

**Published:** 2024-11-13

**Authors:** Pedro Gabriel Senger Braga, Janaína da Silva Vieira, Aline Rachel Bezerra Gurgel, Patricia Chakur Brum

**Affiliations:** ^1^ Laboratory of Cellular and Molecular Exercise Physiology, School of Physical Education and Sport of University of São Paulo, São Paulo, Brazil; ^2^ Clinica Pro-Coracao, São Paulo, Brazil; ^3^ Molecular Oncology Center, Sírio-Libanês Hospital, São Paulo, São Paulo, Brazil; ^4^ Department of Physiology and Biophysics, Institute of Biomedical Sciences of University of Sao Paulo, São Paulo, Brazil

**Keywords:** drug repositioning, solid cancer, β-blockers, statin, survival

## Abstract

Despite advances in cancer treatment, current cancer incidence and prevalence still demand multimodal treatments to enhance survival and clinical outcomes. Drugs used in cardiology, such as β-blockers and statins have gained attention for their potential roles in oncology. This review focused on their possible complementary use in solid tumors, including breast, colorectal, lung, and prostate cancers. The involvement of the autonomic nervous system in promoting tumor growth can be disrupted by β-blockers, potentially hindering cancer progression. Statins, known for their pleiotropic effects, may also inhibit cancer growth by reducing cholesterol availability, a key factor in cell proliferation. We will provide an update on the impact of these therapies on cancer treatment and surveillance, discuss the underlying mechanisms, and explore their effects on the heart, contributing to the growing field of cardio-oncology.

## Introduction

Cancer cases were estimated at 19.3 million, with 10 million deaths globally in 2020. The burden of cancer continues to raise concern, with 28.4 million cases projected by 2040, representing a 47% increase compared to 2020 (Sung et al., 2021). Breast and prostate cancers, followed by lung, bronchus, and colorectal cancers, are the most prevalent across both sexes (Sung et al., 2021). Over the last decade, significant progress has been made in understanding the hallmarks of cancer development and treatment. These advances have improved cancer therapy and extended survival rates. Currently, cancer treatments include surgery, chemotherapy, radiotherapy, immunotherapy, and targeted therapy. The use of existing drugs has also emerged as a promising strategy in the fight against cancer. In this context, drug repositioning involves finding new applications for approved or investigational drugs beyond their original medical indications (Ashburn and Thor, 2004). The advantages of repurposing drugs include time savings, cost-effectiveness, safety, and efficiency, given their well-known pharmacological properties and toxicity profiles (Malik et al., 2023). Especially in the field of cardio-oncology, drug repositioning is emerging as a multifactorial therapy within the cancer continuum, as it may not only prevent or mitigate treatment-related side effects but also improve survival and reduce all-cause mortality (Lee et al., 2020; Attar et al., 2022). In this context, β-blockers and statins have been considered promising repurposing options for cancer treatment due to their clinical benefits and safety. Importantly, these drugs offer overlapping benefits in both cardiovascular and oncology outcomes, as they may minimize cardiovascular complications associated with cancer therapies. For example, the use of β-blockers in breast cancer (BC) treatment has been associated with reduced cardiotoxicity and decreased overall and cardiovascular mortality (Attar et al., 2022; He et al., 2022). Similarly, these benefits extend to statin treatment (Lee et al., 2020; Tailor et al., 2021). Notably, the reduction of low-density lipoprotein cholesterol (LDL-C) plasma concentrations helps to lower the cardiovascular disease (CVD) burden in the cancer population (Mohamed et al., 2024). This is particularly significant since one of the leading causes of death in cancer survivors is CVD (Zaorsky et al., 2017).

Mechanistically, increased sympathetic nervous activity may promote cancer progression (Stavropoulos et al., 2020), which is a target for use of β-blockers. Supporting this, solid tumors have been observed to signal new innervation, attracting nerve growth to the cancer (Li et al., 2022). Regarding statins, solid tumors require more structural support for development, and cholesterol, a structural lipid, may play a role in promoting this growth (Xiao et al., 2023; Halimi and Farjadian, 2022). This study aims to provide a deeper examination and exploration of the potential role of statins and β-blockers in different populations to improve cancer outcomes during treatment.

In this review, we will now explore the impact of β-blockers and statins on overall and disease-free survival in the conventional treatment of breast, colorectal, lung, and prostate cancers. These cancers were selected due to their high prevalence and mortality rates in both sexes globally (Sung et al., 2021), as well as the potential interactions of β-blockers and statins with standard therapies. Our review will encompass data from observational studies, primarily retrospective analyses, and randomized clinical trials within the cancer continuum. Our goal is to provide further insight into their role in oncology, encouraging their consideration as part of integrated cancer treatment strategies.

## β-blockers and statins: the role against breast cancer cardiotoxicity

BC is the most common neoplasia in women and it is markedly characterized by heterogeneous molecular profiles, based on hormonal receptors and human epidermal growth factor receptor-2 (HER2) status. The conventional multimodal therapy used for BC patients includes surgical tumor resection, chemo, and radiotherapy (Loibl et al., 2021) and has successfully resulted in a growing number of survivors. However, this population is at risk of developing treatment-induced CVD compared to healthy controls, especially those treated with anthracycline agents (i.e., doxorubicin, daunorubicin, epirubicin, idarubicin, and mitoxantrone). Cancer survivors who underwent chemotherapy have increased resting heart rate (Lakoski et al., 2015), and higher circulating norepinephrine levels (Nousiainen et al., 2001), which are suggestive of sympathetic overdrive. Because β-adrenergic receptors (β-AR) also may play a role in invasion and cancer progression (Hiller et al., 2020), β-AR antagonists, also called β-blockers, are emerging as novel anticancer therapeutics. For BC therapy, β-blockers are an evolving intersection of the cardiovascular and oncology fields and represent a strategy to counteract treatment-induced cardiotoxicity.

β-blockers are a group of antihypertensive agents with diverse mechanisms of action. Overall, they antagonize adrenergic stimulation of β-AR according to receptor selectivity (β1 and β2-AR), exhibit intrinsic sympathomimetic activity, vasodilating properties, and modulate metabolism (Gorre and Vandekerckhove, 2010). Importantly, only the subtypes β1 and β2-AR encompass the scope of this review.

Because the heart has a larger proportion of β1-AR compared to β2-AR, specifically in a 70/30 ratio (Gorre and Vandekerckhove, 2010), the effect of β-blockade on the heart is heterogeneous. Stimulating β1 leads, cyclic adenosine monophosphate (cAMP)-dependent intracellular pathways, to positive inotropic, chronotropic, lusitropic and dromotropic effects (Triposkiadis et al., 2009); but also, stimulating β2-AR in arterial walls results in vasodilation.

Thus, β-blockers are stratified in “generations” according to their pharmacological properties. The first-generation of β-blockers is the non-selective, blocking both β1 and β2 receptors, such as propranolol. The second-generation agents include β-blockers with higher affinity for β1-AR, which is often referred to as “cardio”-selectivity. Examples include atenolol, metoprolol, bisoprolol and betaxolol. The third generation of β-blockers exhibit varied selectivity for β1-AR (do Vale et al., 2019). They may stimulate β2-AR leading to vasodilation, nitric oxide synthesis and anti-inflammatory properties (Triposkiadis et al., 2009; Bakris, 2009). Additionally, vasodilation may also be mediated by blocking alpha1-AR and activating β3-AR. The third generation β-blockers include agents like pindolol, carvedilol and nebivolol (do Vale et al., 2019).

Many RCTs addressed cardiotoxicity in BC by evaluating left ventricular ejection fraction (LVEF). Considering the cancer *continuum*, at the pre-chemotherapy stage, starting lisinopril and bisoprolol (β_1_-AR antagonist) 24 h before the first cycle of chemotherapy reduced the decline in LVEF and prevented anthracycline-induced cardiotoxicity in women with advanced BC (Wihandono et al., 2021). Endorsing it, LVEF was unchanged in the carvedilol (β_1_/β_2_ -AR antagonist) group but decreased in the control arm (Nabati et al., 2017). During chemotherapy, administering carvedilol was shown to prevent doxorubicin-induced cardiotoxicity compared with a placebo. Beyond LVEF, strain and strain-rate function, which are more sensitive indices to detect early stages of myocardial dysfunction, were examined. Even though these measures worsened in the control group following chemotherapy, they were unchanged in subjects who received carvedilol (Tashakori et al., 2016). Carvedilol administered daily for 6 months during chemotherapy maintained LVEF and fractional shortening within normal limits for all patients before and after anthracycline treatment, indicating a cardioprotective effect (Elitok et al., 2014). Starting candesartan or carvedilol within 1 month after initiating doxorubicin-containing chemotherapy, and maintaining it throughout the treatment might have prevented an early decrease in LVEF (Lee et al., 2021). The MANTICORE 101-Breast Study examined women with HER2-positive early BC on adjuvant therapy with trastuzumab. It was observed that perindopril and bisoprolol protected against cancer therapy-related declines in LVEF; nonetheless, trastuzumab-mediated left ventricular remodeling could not be prevented by this drug protocol (Elitok et al., 2014). Another study showed that β-blockers protect against cardiotoxicity following trastuzumab and anthracycline-containing medication treatment (Gao et al., 2023). Besides, continuous use of β-blockers was associated with a lower heart failure incidence in patients with BC under trastuzumab treatment (Seicean et al., 2013). The concomitant use of β-blockers with angiotensin-converting enzyme receptors was associated with a recovery of LVEF during months 3–12 of adjuvant trastuzumab therapy (Oliva et al., 2012). Using metoprolol (β_1_-AR antagonist) and candesartan concomitantly with epirubicin-based anthracycline therapy increased circulating cardiovascular biomarkers without association with a decline in ventricular function in early BC women. In this same study, troponin levels were attenuated by metoprolol, but not candesartan (Gulati et al., 2017). HER2-positive BC women showed that cardiotoxicity-free survival was longer on both lisinopril and carvedilol treatment compared to placebo (Guglin et al., 2019). Complementing these findings, the Prevention of Cardiac Dysfunction During Adjuvant Breast Cancer Therapy (PRADA) study suggested that a cardioprotective approach might not be required in patients without preexisting CVD (Heck et al., 2021). Regarding the post-chemotherapy stage, one study pointed out higher LVEF in the β-blocker group compared with placebo (Shah et al., 2019).

Summarizing, there is evidence that β-blockers prevent chemotherapy-induced cardiotoxicity and improve or contribute for maintenance of LVEF and better survival. Carvedilol, a third generation β-blocker which has additional alpha-blockade and vasodilating properties, was the most cited intervention used during chemotherapy and led to cardioprotective effects with preservation of LVEF. In addition, the combined use of β-blockers with angiotensin-converting enzyme receptors was associated with LVEF recovery during cancer therapy. Importantly, β-blockers exhibit only minor side effects and may prove an effective, safe, and inexpensive anticancer repurposing strategy, being a feasible alternative to integrate BC treatment.

Another potential drug repurposing candidate for cancer are statins. Statins belong to a class of drugs that may be divided into two types, according to their solubility in the lipidic environment, being either lipophilic or hydrophilic. The solubility may characterize the statins’ capacity to be incorporated into the liver. Lipophilic statins can be incorporated by passive diffusion (i.e., atorvastatin, simvastatin, fluvastatin, and pitavastatin), while hydrophilic statins require active transport (i.e., pravastatin and rosuvastatin) (Minichsdorfer et al., 2022). Statins act by inhibiting 3-hidroxi-3-methyl-glutaril-CoA redutase HMG-CoA reductase, which reduces cholesterol synthesis in the liver, thereby decreasing blood cholesterol carried by LDL-C. The survival rates associated with statin treatment in BC patients may be related to their chemopreventive effects on attenuating cardiotoxicity. The impact of statin treatment on primary and secondary prevention of CVD is well known and it reduces chemotoxicity resulting from BC treatment (Mohamed et al., 2024). In BC patients, the prophylactic use of 40 mg of atorvastatin, a lipophilic statin, for 6 months may prevent cardiac dysfunction resulting from anthracycline-based therapy (Mohamed et al., 2024). Similar results were observed in BC patients under treatment of rosuvastatin, hydrophilic statin (Nabati et al., 2019). Corroborating these data, HER2+ BC patients under statins who were receiving trastuzumab, with or without anthracyclines, displayed a lower risk for cardiotoxicity (Calvillo-Arguelles et al., 2019). A study showed that LVEF did not change with statin treatment, whereas in the placebo groups, it decreased over time (Mohamed et al., 2024; Nabati et al., 2019; Calvillo-Arguelles et al., 2019). In contrast, another study could not endorse the results, and showed that treatment based on 40 mg of atorvastatin did not improve cardiovascular function. The possible reason for this may be the heterogeneity of cancers addressed, since the study included breast, lymphoma, leukemia, sarcoma, and thymoma; also, these patients were more prone to develop complications, with a higher risk for treatment-induced toxicity (Thavendiranathan et al., 2023). Like β-blockers, statins seem safe and beneficial against cardiotoxicity. In addition, they might have a positive effect on overall survival in cancer patients, as discussed below. Because cholesterol may serve as substrate for growth and development of cancer cells; and considering that statins inhibit cholesterol production, placing these drugs as an adjuvant treatment hold promise to improve survival (Xiao et al., 2023; Halimi and Farjadian, 2022).

## β-blockers and breast cancer: survival analysis

Retrospective observational studies indicated that β-blockers improved BC-related clinical outcomes, regardless of β-blocker selectivity (Botteri et al., 2013; Choy et al., 2016; Gillis et al., 2021; Powe et al., 2010; Sørensen et al., 2013). A meta-analysis study that addressed β-blockers, angiotensin-converting enzyme inhibitors (ACEi) and angiotensin II receptor blockers (ARB) use showed that β-blockers improved BC specific survival for women treated at the time or in the year across BC diagnosis; however, the association of ACEi/ARB did not yield to disease free and overall survival. In this study, between-study heterogeneity was assessed to investigate outcomes for propranolol vs other β-blockers. The antiangiogenetic and antimetastatic property of β-blockers, in particular propranolol, was endorsed (Raimondi et al., 2016). Another meta-analysis, which did not specify β-blocker selectivity, found that β-blocker use after diagnosis but not before diagnosis is beneficial for the survival of cancer patients (Zhong et al., 2016). The positive effects of β-blockers on survival were confirmed also for patients with triple-negative BC (TNBC), with the most prevalent drugs used being selective β-blockers, mainly metoprolol; followed by atenolol (Melhem-Bertrandt et al., 2011). BC-specific mortality and presenting with advanced tumors were significantly lower for propranolol users (Barron et al., 2011). A lower prevalence of cardiovascular toxicity regardless of the time of initiation of β-blockers (Martins Carvalho et al., 2023) and reduced risk of cardiovascular mortality upon using metoprolol, atenolol, and carvedilol Tabassum et al. (2024) have been reported. The ROSE/TRIO-012 Study examined survival and clinical benefits in patients with HER2-negative advanced BC treated with docetaxel in combination with ramucirumab or placebo. The use of β-blockers was associated with BC specific survival only in patients with TNBC, with hazard ratios of 0.66 (95% CI: 0.47–0.91) for use of any type of β-blocker, 0.24 (95% CI:0.06–0.96) for use of non-selective β-blocker, and 0.71 (95% CI:0.51–0.99) for use of selective β-blockers (Løfling et al., 2022).

However, other studies failed to confirm associations between β-blocker therapies and improved outcomes. Studies that stratified the population between β-blocker users and non-users showed that the observed poor disease-free survival did not endorse β-blockers antitumor effects (Cardwell et al., 2016; Kim et al., 2017; Li et al., 2020; Sakellakis et al., 2014). Likewise, another study that was not able to address the selectivity of β-blockers could not point to improved mortality deaths and recurrence (Li et al., 2020). Also, using perioperative β-blockers (selectivity not specified) did not improve cancer-specific survival before and after index surgical resection for the breast (Musselman et al., 2018). Contrarily, β-blockers were linked to poorer progression-free and overall survival based on their use before cancer diagnosis, with the most prevalent prescribed drugs being atenolol, followed by propranolol and another β-blocker (Shah et al., 2011). Using propranolol or non-selective β-blockers was not associated with improved survival (Cardwell et al., 2016). Corroborating, it was shown that trastuzumab concomitantly with any dose of a β-blocker (propranolol, carvedilol, atenolol, or bisoprolol) potentially had negative outcomes in women with HER2-positive advanced BC (Hsieh et al., 2023). Nonetheless, in a study that compared β-blocker users to non-users, death was associated with short-term (0–3 months) β-blocker therapy, indicating a protective effect associated only with long-term use (i.e. 3 years +) (Scott et al., 2022).

Overall, RCT studies showed that using β-blockers was associated with cardioprotective effects for women with BC receiving anthracyclines over the cancer *continuum* and showed promising results for improved survival in BC, regardless of the selectivity of the drug, when reported. In general, results are summarized in Table 1.

**TABLE 1:** Effect of β-blockers on breast cancer survival.

Author, year	Survival outcome
Botteri et al. (2013)	↑
Choy et al. (2016)	↑
Gillis et al. (2021)	↑
Powe et al. (2010)	↑
Sørensen et al. (2013)	↑
Raimondi et al. (2016)	↑
Zhong et al. (2016)	↑ - Only when used after cancer diagnosis
Melhem-Bertrandt et al. (2011)	↑
Barron et al. (2011)	↑
Tabassum et al. (2024)	↑
Løfling et al. (2022)	↑ - Only in TNBC
Cardwell et al. (2016)	↔
Kim et al. (2017)	↔
Li et al. (2020)	↔
Sakellakis et al. (2014)	↔
Musselman et al. (2018)	↔
Shah et al. (2011)	↓
Hsieh et al. (2023)	↓
Scott et al. (2022)	↓ Short-term use (0–3 months) ↑ Long-term use (>3 years)

↑: increase; ↓: decrease; ↔: unchanged.

Retrospective studies, however, were unclear concerning the use of β-blockers in the BC approach. These conflicting outcomes might be explained by methodological limitations. Previous studies fail reporting the time of medication use. Besides, there was scarce information on between β-blockers and concurrent use of other medication for chronic diseases, such as diabetes or dyslipidemia. Additionally, because these studies normally address data of women from Europe or North America, the association between β-blockers use and prognosis in BC should also consider women with other ethnicities because BC incidence and prevalence differ among populations globally. It is crucial to point out that most retrospective studies were unable to evaluate the selectivity of β-blockers and how it would yield to different prognosis since this data were rarely retrieved. So, it is recommended to investigate future studies to identify the doses, exposure, and selectivity of β-blockers repurposed in cancer.

## Statins and breast cancer: survival analysis

Similarly to β-blockers, statins are widely known for their cholesterol-lowering effects, have also garnered attention for their possible benefits in cancer therapy. The use of statins is mainly directed at the control of blood cholesterol concentrations. Total cholesterol levels are associated with BC mortality, and so, its reduction and control via statin treatment may directly impact the patient’s survival (Murto et al., 2023). Beyond the recurrence risk (Jia et al., 2023), improvement of BC survival was demonstrated in reviews and meta-analysis (Jia et al., 2023; Wu et al., 2015; Beckwitt et al., 2018), but also in cohort studies (Li et al., 2019; Borgquist et al., 2019). Data from 1.3 million of patients from UK Biobank show the associative protector role of statin on the prognosis of BC patients. Also, statin appears to have the ability to convert a cold to hot tumor (Li et al., 2024). It means the increased capacity to respond to anticancer therapies. Corroborating with these data, women who were receiving statins for 6 months before BC diagnosis presented a lower risk of all-cause mortality compared with non-users, which was mainly attributed to cancer-related rather than cardiovascular death. Moreover, the reduction of cancer-related deaths was time-dependent on the statin treatment (Chang et al., 2023). Endorsing it, retrospective studies showed that using statins for more than 5 years was associated with higher overall and disease-free survival in comparison to non-statin users in BC, regardless of neoplasia type (Li et al., 2019; Sim et al., 2022).

In the Austrian Breast and Colorectal Cancer Study Group (ABCSG) trial 18, postmenopausal women with hormone receptor-positive BC who were receiving adjuvant denosumab or placebo were examined. In the treatment arm, which was taking hydrophilic statin, disease-free survival was worse in comparison to non-users; however, after correcting for confounders such as age, ethnicity, and body-mass index, and others, the hydrophilic statin’s detrimental effect was attenuated. On the other hand, lipophilic statins, such as atorvastatin, simvastatin, fluvastatin and pitavastatin, did not lead to any effects on survival (Minichsdorfer et al., 2022).

Despite scarce data, statins-based long-term treatment appears to be beneficial for BC women, especially before diagnosis, according to Table 2. Further studies should explore the clinical outcomes regarding statin solubility. For now, new strategies need to be considered to replace statins as primary prevention in the BC scenario. Statins off-label repositioning is attractive within the BC context because they may mitigate chemotherapy-related cardiotoxicity and display some benefits on overall survival.

**TABLE 2:** Effect of statins on breast cancer survival.

Author, year	Survival outcome
Murto et al. (2023)	↑
Jia et al. (2023)	↑
Wu et al. (2015)	↑
Beckwitt et al. (2018)	↑
Li et al. (2019)	↑
Borgquist et al. (2019)	↑
Chang et al. (2023)	↑
Sim et al. (2022)	↑
Minichsdorfer et al. (2022)	↔

↑: increase; ↓: decrease; ↔: unchanged.

Another neoplasia that raises concern is colorectal cancer (CRC), which is increasingly affecting younger patients, with rising incidence and mortality rates.

## β-blockers and colorectal cancer

CRC is a significant health concern worldwide, characterized by its high incidence and mortality rates (Bray et al., 2024). Despite advancements in treatment strategies, managing CRC remains challenging, particularly in the advanced stages of the disease (Cercek et al., 2020; Chen et al., 2023).

Several preclinical studies have provided insights into the potential benefits of β-blockers in CRC (Mohammadpour et al., 2021; Qiao et al., 2019; Qiao et al., 2021). Despite promising preclinical data, clinical evidence regarding the efficacy of β-blockers in CRC remains somewhat limited and inconclusive. Cui and colleagues showed an increase in overall survival in patients with CRC using β-blockers regardless of chemo or radiotherapy regimen, time of β-blocker use, and stage of the disease (Cui et al., 2019). However, many other retrospective studies have failed to replicate these findings (Musselman et al., 2018; Shah et al., 2011; Ekestubbe et al., 2022; Hicks et al., 2013; Holmes et al., 2013; Numbere et al., 2017; Zhang et al., 2022).

Interestingly, some studies that stratified patients by stage of the disease show promising results. Balakrishnan et al. showed that the use of β-blockers is associated with reduced mortality in patients with stage I-III of CRC (Balkrishnan et al., 2021). Jansen and colleagues evaluated CRC patients of different stages of the disease, and only patients at stage IV showed benefits from the use of β-blockers (Jansen et al., 2012; Jansen et al., 2014).

The type of primary cancer therapy seems to also alter β-blocker benefits. Giampieri et al. showed that the association of β-blockers and chemotherapy in metastatic CRC patients led to improvement in overall survival, but when the patients were also treated with bevacizumab, the opposite effect was observed (Giampieri et al., 2015). However, the number of patients receiving therapy in this study was small (nine) and the conclusion might not be representative of the population. In a larger study with metastatic CRC patients receiving chemotherapy and immune checkpoint inhibition, β-blockers showed a beneficial effect on overall survival and progression-free survival (Kocak et al., 2023).

In the context of CRC, β-blocker-based treatment led to mixed results, with studies reporting benefits, whereas others reporting neutral outcomes, according Table 3. However, no study pointed out the worsening in patient’s condition upon β-blockers use. Therefore, we endorse that more studies are needed to clarify the real effect of β-blocker on CRC survival.

**TABLE 3:** Effect of β-blocker on colorectal cancer survival.

Author, year	Survival outcome
Mohammadpour et al. (2021)	↑
Qiao et al. (2019)	↑
Qiao et al. (2021)	↑
Cui et al. (2019)	↑
Musselman et al. (2018)	↔
Shah et al. (2011)	↔
Ekestubbe et al. (2022)	↔
Hicks et al. (2013)	↔
Holmes et al. (2013)	↔
Numbere et al. (2017)	↔
Zhang et al. (2022)	↔
Balkrishnan et al. (2021)	↑
Jansen et al. (2012)	↑
Jansen et al. (2014)	↑
Kocak et al. (2023)	↑

↑: increase; ↓: decrease; ↔: unchanged.

## Statins and colorectal cancer

Statins appear to be beneficial and safe for use during CRC treatment. In combination with other non oncology-related drugs, such as aspirin and anti-inflammatory, statins may improve patient survival (Bardou et al., 2010). In cohort studies, statin treatment displays interesting results. When initiated before CRC diagnosis, but not after, statins were associated with improved cancer-specific survival (Gray et al., 2016). In another cohort with a mean duration of 5.6 years, statin use was associated with a lower risk of CRC-related mortality and, also, all-cause mortality (Sun et al., 2023).

Conversely, the effects of statin treatment on survival could not be confirmed. A multi-center study with 269 patients previously treated for metastatic CRC, showed that 7 months of using 40 mg of simvastatin did not improve survival (Lim et al., 2015). Corroborating, two trials that investigated the mutation of KRAS protein, which is a widely studied oncogene associated with malignancies, found no impact of statins on survival in metastatic CRC patients, even though statins often inhibit the expression of KRAS phenotype (Krens et al., 2014; Ng et al., 2011).

Results are presented in Table 4, but as previously observed with BC patients, the association between statin use and survival benefits appears to be associated with the exposition of drugs during the time. Long-term use of statins, mainly, when started before cancer diagnosis (Sun et al., 2023; Dobrzycka et al., 2020; Siddiqui et al., 2009) leads to better clinical outcomes. Thus, when started before or even during treatment, statins have the potential to prevent side effects of CRC treatment (Dobrzycka et al., 2020). Additional studies, mainly retrospective, need to better investigate the time-dependent effect of statin treatment on CRC to reinforce the drug-repositioning on primary prevention. Interestingly, none of the trials reported impairments in patient’s health following statin treatment. Based on the above, it can be suggested that statins are safe and do not lead to significant adverse effects in CRC treatment.

**TABLE 4:** Effect of statin on colorectal cancer survival.

Author, year	Survival outcome
Gray et al. (2016)	↑ - When initiated before cancer diagnosis
Sun et al. (2023)	↑
Lim et al. (2015)	↔
Krens et al. (2014)	↔
Ng et al. (2011)	↔

↑: increase; ↓: decrease; ↔: unchanged.

## β-blockers and prostate cancer

Prostate cancer (PC) is the second most common neoplasia among men and the fifth leading cause of death (Sung et al., 2021). β-blockers are particularly interesting to be used during PC therapy because the activation of β-adrenergic receptors is related to angiogenesis, proliferation, and migration of tumor cells (Malik et al., 2023). Although research suggests a role for these receptor pathways in prostate neoplasia, outcomes on β-blockers and prostate remain unclear.

The following evidence was retrieved from observational studies using population-based cancer registries and hospital electronic medical records. Men under sotalol, (non-selective blocker of β_1_ with antiarrhythmic properties), had a decreased risk of PC according to the duration of sotalol use (Kaapu et al., 2015). β-blocker treatment was associated with a 10% lower risk for all PC in models adjusted for age and race. Importantly, this therapeutic benefit was lost when adjusting it for the history of CVD (Rodriguez et al., 2009). In a study with ethnically diverse men, participants were stratified according to the risk of PC. Atenolol (β_1_-AR selective antagonist) was associated with a 38% reduction in odds of incident PC compared to men not taking a β-blocker. In addition, taking atenolol for 3–5 years was associated with a substantial reduction in intermediate and high-risk disease (Zahalka et al., 2020). β-blockers were associated with reduced PC-specific mortality, but no effect was reported on all-cause mortality (Grytli et al., 2013; Lu et al., 2015).

Conversely, the Finnish Randomized Study of Screening for Prostate Cancer showed no general protective effects against PC death with digoxin usage, sotalol, and antiarrhythmic drugs in general, before or after PC diagnosis (Kaapu et al., 2016). Hazard ratios of mortality outcomes associated with post-diagnostic use of β-blockers in men diagnosed with non-metastatic PC revealed that β-blockers, regardless of selectivity, were not associated with a decreased risk of PC and all-cause mortality (Assayag et al., 2014). Even in patients at high-risk PC on androgen deprivation therapy, using any β-blocker was not associated with improved survival (Posielski et al., 2021). In addition, the use of antihypertensive drugs was associated with an increased risk of PC. However, because the authors addressed distinct categories of antihypertensive drugs separately and in combination, this outcome may be related to underlying hypertension (Siltari et al., 2018).

We could not retrieve RCT data in our search. Taken together, the retrospective studies on PC mainly addressed outcomes such as risk and mortality of cancer and shared conflicting results, according to Table 5. While studies point out that β-blocker-based treatment was associated with a decreased risk of developing PC, others report no protective effects for mortality and risk; and one study positively associated β-blocker and PC. Future research will be required to determine whether β-blockers have differential effects as a function on patients’ survival.

**TABLE 5:** Effect of β-blocker on prostate cancer survival.

Author, year	Survival outcome
Grytli et al. (2013)	↑
Lu et al. (2015)	↑
Kaapu et al. (2016)	↔
Assayag et al. (2014)	↔
Posielski et al. (2021)	↔

↑: increase; ↓: decrease; ↔: unchanged.

## Statins and prostate cancer

The risk of fatal PC was lower in statin users than in non-users (Craig et al., 2022). Adopting statin therapy before PC diagnosis did not impact survival, however, when starting it following diagnosis, statins were associated with decreased PC-related mortality. The decreased risk was dose-dependent and observed especially among patients undergoing hormone therapy (Murtola et al., 2017). Statin outcomes are often linked to a better prognosis, regardless of whether it is hydrophilic or lipophilic (Cao et al., 2023). A study reported good prognosis and overall survival in men with castration-resistant metastatic PC (Hamilton et al., 2021). In general, prospective studies endorse positive results concerning the effect of statin treatment on PC mortality (Gutt et al., 2010; Joentausta et al., 2019; Kollmeier et al., 2011; Van Rompay et al., 2019; Wu et al., 2019; Yu et al., 2014). These benefits are independent of the type of treatment, such as surgery or hormone therapy, and cancer grade. Additionally, similar to findings in other previously described cancers such as CRC and BC, patients who are taking statins before diagnosis experience a greater beneficial effect against PC (Yu et al., 2014).

In a study with over one thousand patients who were initiating androgen deprivation therapy (ADT), statin therapy was related to reduced overall mortality (Hamilton et al., 2021). Likewise, statin treatment also had positive effects on a population-based cohort study that involved 4,428 men and had a 6.3-year follow-up. The treatment initiated after ADT was associated with improved PC prognosis and survival (Peltomaa et al., 2021).

Statin treatment is widely supported in the literature as a strategy against PC-related and overall mortality, as shown in Table 6. Statins prescription needs to be reconsidered by clinicians aiming for better cancer-related prognosis but also in the environment of primary prevention, such as controlling risk factors for CVD.

**TABLE 6:** Effect of statin on prostate cancer survival.

Author, year	Survival outcome
Craig et al. (2022)	↑
Murtola et al. (2017)	↑ - Only after taking after diagnosis
Cao et al. (2023)	↑
Hamilton et al. (2021)	↑
Gutt et al. (2010)	↑
Joentausta et al. (2019)	↑
Kollmeier et al. (2011)	↑
Van Rompay et al. (2019)	↑
Wu et al. (2019)	↑
Yu et al. (2014)	↑
Peltomaa et al. (2021)	↑

↑: increase; ↓: decrease; ↔: unchanged.

## β-blockers and lung cancer

Lung cancer (LC) remains one of the most prevalent and deadly forms of cancer worldwide, with a high mortality rate despite advances in treatment (Bray et al., 2024). It often presents in advanced stages, challenging successful treatment (Nilsson et al., 2020). However, recent research has shed light on potential adjunct therapies that could improve overall survival rates. In this sense, β-blockers, commonly used to manage hypertension and heart-related conditions (Nilsson et al., 2020) can be a promising adjuvant therapy for cancer. Several studies have suggested a potential link between β-blocker use and improved overall survival in LC patients. One hypothesis is that β-blockers may exert anti-tumor effects by inhibiting signaling pathways involved in cancer progression and metastasis (Schuller and Al-Wadei, 2012; Yan et al., 2023). Additionally, β-blockers might mitigate the harmful effects of stress on tumor growth, as stress hormones can promote tumor development and resistance to treatment (Yan et al., 2023).

Despite promising preliminary evidence, the relationship between β-blockers and LC outcomes remains complex and not fully understood. Musselman et al. (2018) performed a retrospective analysis comparing patients who were exposed to β-blockers and those who were not before and after undergoing surgical resection for breast, lung, and colorectal cancer. Despite the extensive scope of this population-based study, no correlation was found between perioperative β-blocker exposure and enhanced cancer-specific survival rates in individuals with breast, lung, or CRC (Musselman et al., 2018). These results corroborate another retrospective study that evaluated perioperative the use of β-blockers in the overall survivor and progression-free survival (Cata et al., 2014). Other retrospective studies have explored the correlation between β-blocker usage and survival in LC. However, findings from these studies indicate that the administration of β-blockers before and after diagnosis did not change the overall survival of LC patients (Holmes et al., 2013; Coelho et al., 2020; Udumyan et al., 2020; Weberpals et al., 2017). Interestingly, studies comparing the use of β-blockers with some specific therapies have shown promising results. Wang et al. (2013) showed that the incidental use of β-blocker in patients with non-small cell lung cancer (NSCLC) that did radiotherapy is associated with progression-free survival, distant metastasis-free survival, disease-free survival, and overall survival. These results corroborate the findings of Chaudary and colleagues, who observed in a retrospective study the association of β-blockers with chemo and radiotherapy in patients with NSCLC led to increased overall survival and distant metastasis-free survival (Chaudhary et al., 2019).

In general, studies have reported conflicting results, as shown in Table 7, highlighting the need for further research to elucidate the mechanisms underlying any potential benefits, especially through prospective studies, since almost all studies published so far are retrospective. Factors such as dosage, duration of use, and selectivity of β-blocker used and combination with other cancer therapies may also influence outcomes, calling for careful consideration in future studies. If β-blockers indeed prove to enhance overall survival in LC patients, they could represent a readily available and affordable adjunct therapy with the potential to positively impact patient care. However, rigorous clinical trials are needed to confirm these findings and establish clear guidelines for their use in the oncological setting. Similar results were observed in respect to statin and LC, which are necessary for more studies, and also, with the combination of both drugs.

**TABLE 7:** Effect of β-blockers on lung cancer survival.

Author, year	Survival outcome
Musselman et al. (2018)	↔
Cata et al. (2014)	↔
Holmes et al. (2013)	↔
Coelho et al. (2020)	↔
Udumyan et al. (2020)	↔
Weberpals et al. (2017)	↔
Wang et al. (2013)	↑
Chaudhary et al. (2019)	↑

↑: increase; ↓: decrease; ↔: unchanged.

## Statins and lung cancer

Statins are under-prescribed for patients with LC. Importantly, in LC patients, atherosclerotic disease is the leading cause of death (Tailor et al., 2021). Therefore, prevention of dyslipidemia and its adequate treatment are important strategies to prevent atherosclerotic CVD in cancer patients, and statin adherence is associated with reduced all-cause, cancer-related and cardiovascular mortalities (Lee et al., 2020).

Drug repositioning is already widely adopted in LC treatment. Using diverse drugs such as metformin, aspirin, and statin in combination is protective against LC mortality. In addition, statin alone reduces LC mortality in a dose-dependent manner (Kang et al., 2021). In the first line treatment, with epidermal growth factor receptor kinase inhibitors, statin was associated with prolonged life survival in LC patients (Nguyen et al., 2020).

Retrospectively, the post-diagnosis treatment with simvastatin and atorvastatin was associated with extended survival in NSCLC patients (Ung et al., 2018). In general, statin reduced rates of cancer-specific mortality, especially simvastatin, which is a lipophilic drug (Cardwell et al., 2015). However, in a RCT addressing patients with both NSCLC and SCLC, statin therapy did not appear to improve prognosis. In a phase II trial with afatinib plus 40 mg/day of simvastatin or afatinib alone, with 68 previously treated patients with advanced non-adenocarcinomatous NSCLC, it was not possible to establish additional clinical benefits for using statins in survival (Lee et al., 2017). In the phase II trial with SCLC, which examined the effectiveness of irinotecan plus cisplatin with and without 40 mg/day of simvastatin in 125 patients, survival was not improved (Lee et al., 2023). Another phase III, randomized, double-blind, placebo-controlled trial with treatment of 40 mg/day of pravastatin added to first-line standard chemotherapy (etoposide plus cisplatin or carboplatin) in SCLC, included 846 patients. Despite safety, pravastatin treatment did not result in better survival (Seckl et al., 2017). Conversely, in another RCT, simvastatin in combination with gefitinib improved survival in NSCLC patients in comparison to those who were not receiving statin treatment (Han et al., 2011).

Overall, clinical trials do not support the effectiveness of statins during LC treatment on survival, according to Table 8, although they are safe for prescription. Depending on the cancer stage and differences in protocol treatment, it is suggested that statins do not aggregate benefits for LC patients; however, concerning overall survival, statins might play a role in reducing CVD-related mortality.

**TABLE 8:** Effect of statin on lung cancer survival.

Author, year	Survival outcome
Kang et al. (2021)	↑
Nguyen et al. (2020)	↑
Ung et al. (2018)	↑
Cardwell et al. (2015)	↑
Lee et al. (2017)	↔
Lee et al. (2023)	↔
Seckl et al. (2017)	↔
Han et al. (2011)	↑

↑: increase; ↓: decrease; ↔: unchanged.

## Other considerations and future perspectives

This study has limitations. Some studies did not fully detail study design, type of drug (i.e., selectivity, solubility) and dose used, mainly when it comes to retrospective studies. Both drugs, β-blockers, and statins, have potential to modulate prognosis in different cancer populations. The combined use of different β-blockers and statins appear to be effective during the chemo- and radiotherapy treatment, regardless of their pharmacological class, as they also improve survival in cancer patients. Addressing alternative interventions that incorporate the combination of β-blockers and statins may enlighten the understanding on how to achieve better survival and disease-free survival.

Regarding side effects, both drugs may lead to undesirable consequences, although they tend to be mild, and few patients report them. Depending on the dose, class, and generation, statins are known to cause myalgia, which may lead patients to discontinue treatment (Selva-O’Callaghan et al., 2018). Similarly, β-blockers may lead to hypotension, resulting in dizziness and weakness (Koracevic et al., 2022), especially when the dose is not adequately adjusted for the patient. These side effects can affect treatment for dyslipidemia and hypertension, preventing patients without these chronic conditions from benefiting from the anticancer effects of the medications studied. It is important to note that these drugs are generally safe for long-term use worldwide. However, we cannot overlook the cytotoxic effects of β-blockers, which can disrupt healthy cells (Kavakcioglu Yardimci et al., 2021). While these effects may seem negative, they can enhance the ability to induce apoptosis in cancer cells. Additionally, statin therapy appears to improve the effectiveness of chemotherapy by increasing drug concentration within the cells (Ahmadi et al., 2018), suggesting a potential for repurposing these drugs for cancer treatment.

## Conclusion

Using β-blockers and statins are safe during breast, colorectal, lung, and prostate cancers treatment. β-blockers seem to lead to better clinical outcomes for breast cancer, whereas statins were positively associated with greater outcomes in breast, colorectal and prostate treatment, with additional benefits for patients with PC. Much of the intersection between CVD and the cancers discussed in this review share similarities in predisposing risk factors, such as hypertension and dyslipidemia. Thus, controlling these factors either with β-blockers and statins use and/or changes in lifestyle, is a relevant strategy for better cancer survival. As in the treatment of chronic conditions, oncological disease also requires continuous management, so the benefits of being exposed enough to complementary drugs can be translated into clinical outcomes. Therefore, long-term use of β-blockers and statins may influence a better prognosis in cancer survival.

In respect to future directions, the safety, low-cost and effectiveness observed with the use of β-blockers and statins should encourage future trials, which need to enrolled patients with chronic disease and observe, during a follow, the hypothesis if the β-blockers and statin treatment and the ability to control the disease, such blood pressure and LDL-C concentrations, may influence cancer diagnosis and outcome. In addition, future reports need to detail the drug dosage, time of treatment and different classes of β-blockers and statins. Also, though by Mendelian randomization study, a determinant factor is understanding the role of living with these risk factors for a long time may be mandatory, or not, for the survival in the cancer cases.
